# Primary cardiac lymphoma: a clinicopathological study of 121 cases

**DOI:** 10.3389/fonc.2024.1509100

**Published:** 2025-01-07

**Authors:** Shuhui Zhuang, Liudi Chang, Xiaoxi Feng, Weiwen Hu, Zhaobo Yang, Yuanyuan Zhang

**Affiliations:** ^1^ Department of Hematology, Linyi People’s Hospital, Linyi, Shandong, China; ^2^ School of Clinical Medicine, Shandong Second Medical University, Weifang, Shandong, China; ^3^ Spine surgery, Linyi People’s Hospital, Shandong University, Linyi, Shandong, China

**Keywords:** primary cardiac lymphoma, diffuse large B-cell lymphoma, diagnosis, treatment, prognosis

## Abstract

**Background:**

Primary cardiac lymphoma (PCL) is an exceedingly uncommon type of lymphoma that primarily affects the heart and/or pericardium, or manifests through cardiac symptoms due to myocardial infiltration. The infrequency of PCL, coupled with its non-specific clinical presentations, often complicates early diagnosis. This study aims to fill the existing gap in clinical knowledge regarding PCL by detailing a case of PCL and examining its clinical features, auxiliary examinations, treatment approaches, and prognostic outcomes, thereby facilitating early detection and enhancing patient care.

**Methods:**

A thorough search of the PubMed and Chinese National Knowledge Infrastructure (CNKI) database was performed using keywords “heart” and “lymphoma” or “primary cardiac lymphoma”. This search encompassed publications from January 1, 2014, to November 1, 2024.

**Results:**

The review included 121 cases. These cases usually present with atypical symptoms, mainly circulatory and respiratory, including chest tightness, dyspnea, and edema, along with occasional neurological and gastrointestinal symptoms. Echocardiography served as the primary diagnostic method in 92.6% of cases, while a definitive diagnosis was achieved through pathological examination in all cases (100%). Treatment strategies predominantly included surgical intervention (44.6%) and chemotherapy (76.0%). Although surgery did not have a significant effect on survival rates, chemotherapy proved to be critical in improving patient survival.

**Conclusions:**

PCL, which arises in the cardiac or pericardial areas, is generally associated with a poor prognosis. It is essential for clinicians to develop a greater awareness and understanding of the characteristics of PCL to enhance early diagnosis. The timely initiation of chemotherapy is vital for improving survival rates and the overall quality of life for patients with PCL.

## 1 Introduction

Primary cardiac lymphoma (PCL) is a very rare lymphoma that primarily affects the heart and/or pericardium and causes cardiac symptoms due to myocardial infiltration. Early clinical diagnosis is challenging due to the lack of specificity in early clinical symptoms and ancillary testing, and the survival of PCL patients is restricted to a few months if not treated early and effectively. It is therefore critical to investigate the clinical aspects of PCL patients and develop more effective auxiliary diagnostics. This research presents one case of PCL and compares our findings to those described in the literature. To characterize the clinicopathological characteristics, management, and outcome of PCL patients at home and abroad, data from 121 affected individuals were analyzed retrospectively.

## 2 Materials and methods

### 2.1 Data retrieval and methodology

**Figure 1:**
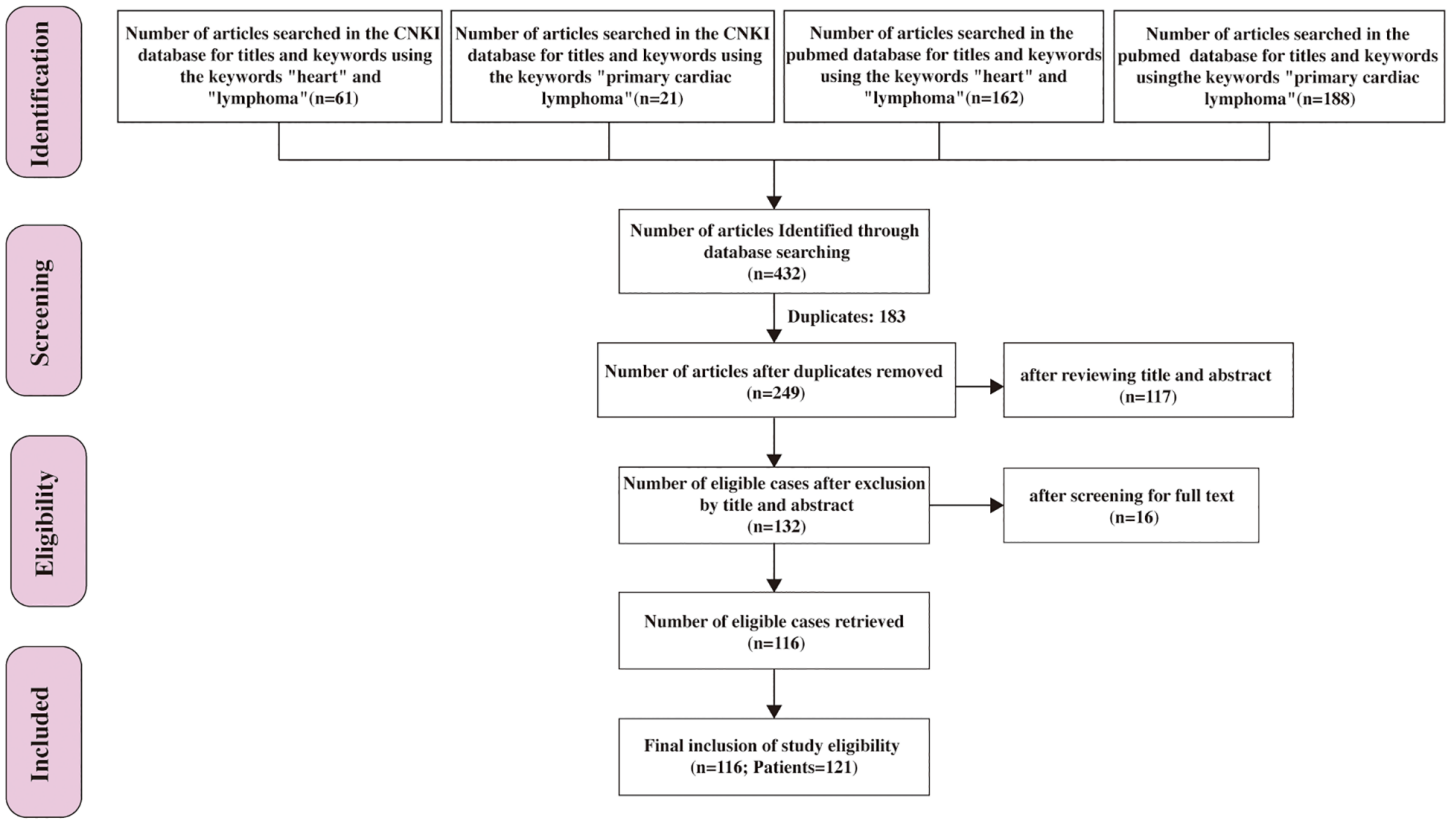
Flow chart depicting the systematic review process utilized in this study.

432 papers were retrieved through a search of the PubMed and Chinese National Knowledge Infrastructure (CNKI) database using the keywords “heart” and “lymphoma” or “primary cardiac lymphoma”. After excluding 183 duplicate entries, 249 articles published between January 1, 2014, and November 1, 2024, were identified. Following a title and abstract screening, 132 papers underwent full-text review. Ultimately, 116 papers were included in this systematic review, consisting of 121 PCL patients, as illustrated in **Figure 1**.

The inclusion criteria were as follows: (1) Diagnosis of PCL established according to the 2015 WHO Classification of Cardiac and Pericardial Tumors (1) under any of the following conditions: i) lymphoma originating in the heart or pericardium; ii) lymphoma presenting with cardiac-related symptoms at initial presentation; or iii) lymphoma primarily manifesting as a cardiac mass. The literature also defines PCL as lymphoma diagnosed in the heart and/or pericardium at initial diagnosis or presenting with cardiac symptoms due to myocardial infiltration by lymphoma, potentially with metastatic manifestations (2–4); (2) No age or gender restrictions were applied; (3) Absence of other serious comorbidities leading to event termination; and (4) In cases of repeated reports, only the earliest published cases were included. Based on the Lugano 2014 criteria for evaluating the efficacy of lymphoma treatment, therapeutic efficacy is assessed using specific standards for patients undergoing positron emission tomography-computed tomography (PET-CT) scans and those who do not. For patients undergoing PET-CT scans, the evaluation is based on the Deauville scoring system. A complete response (CR) is defined as a Deauville score of “1 to 3,” with or without a residual mass, while a partial response (PR) is characterized by a Deauville score of “4 or 5,” accompanied by a reduction in 18F-fluorodeoxyglucose (18F-FDG) uptake compared to baseline, with residual lesions of any size. The Deauville scoring criteria further specify that a score of 1 indicates complete disappearance of tumors, a score of 2 reflects 18F-FDG uptake in the lesion that is less than or equal to the mediastinal blood pool, and a score of 3 indicates 18F-FDG uptake in the lesion that is greater than the mediastinal blood pool but less than or equal to the liver blood pool. A score of 4 represents 18F-FDG uptake in any lesion that is mildly or moderately increased compared to the liver blood pool, while a score of 5 signifies 18F-FDG uptake in any lesion that is significantly increased compared to the liver blood pool (SUVmax > 2 times the liver blood pool) or the appearance of new lesions. For patients who do not undergo PET-CT scans, therapeutic efficacy is deemed effective if there is a reduction or disappearance of target lesions, alleviation of clinical symptoms, or echocardiographic evidence showing that patients with prior significant pericardial effusion exhibit no notable fluid accumulation following puncture and drainage. This comprehensive framework provides a standardized approach to evaluating therapeutic responses in lymphoma patients, ensuring consistency and objectivity in clinical assessments.

### 2.2 Statistical analysis

Statistical analysis was performed using SPSS version 27.0. Quantitative data were expressed as mean ± standard deviation or median ± interquartile range, while qualitative data were presented as case numbers and percentages.

## 3 Results

### 3.1 General information

A total of 121cases met the inclusion criteria. The study included 72 males (59.5%) and 49 females (40.5%), with a male-to-female ratio of 1.435:1. The ages of the patients ranged from 11 to 92 years, with a median age of 62.

### 3.2 Site of onset and clinical manifestations

**Figure 2:**
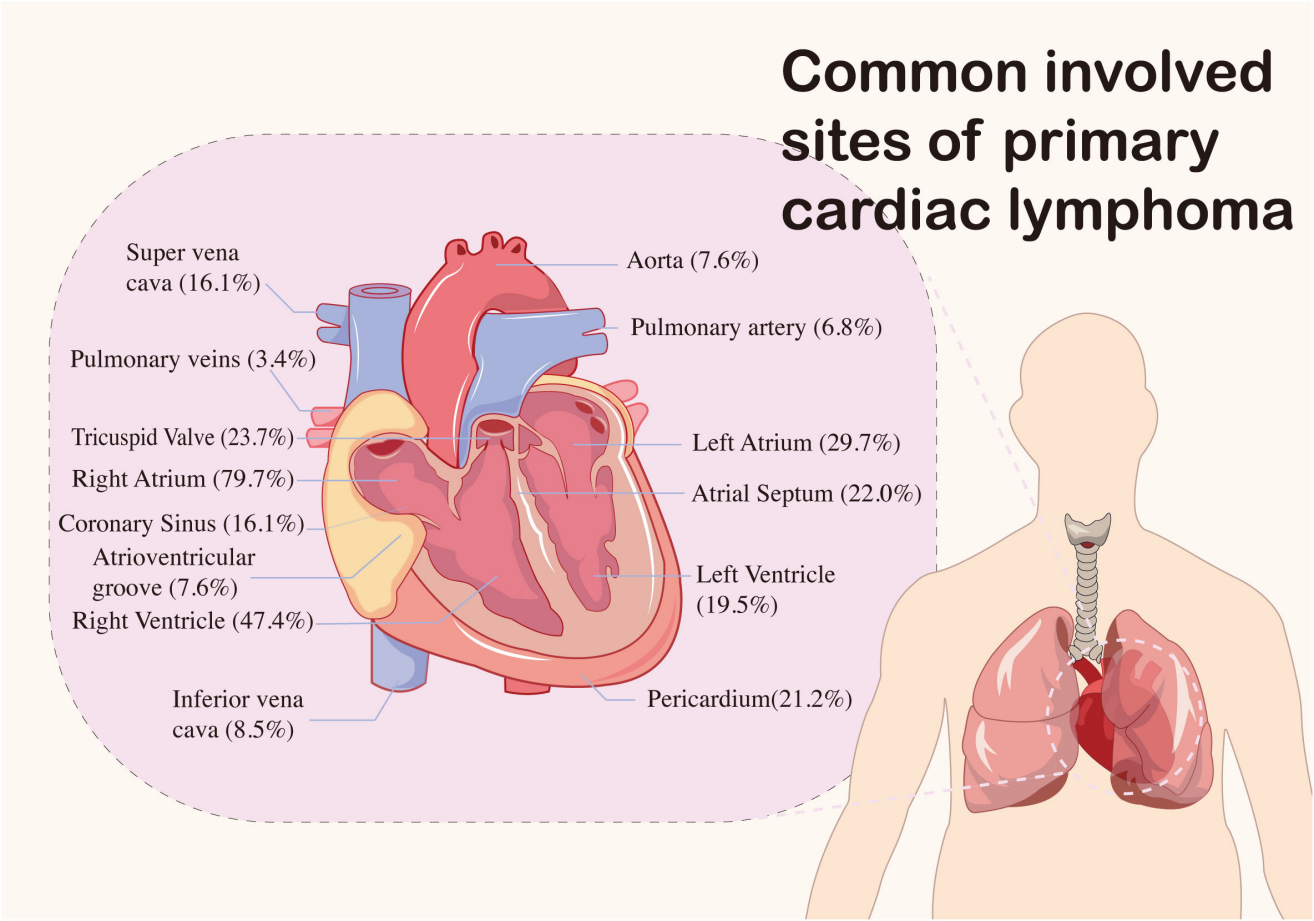
Common sites of primary cardiac lymphoma in 121 cases.

**Figure 3:**
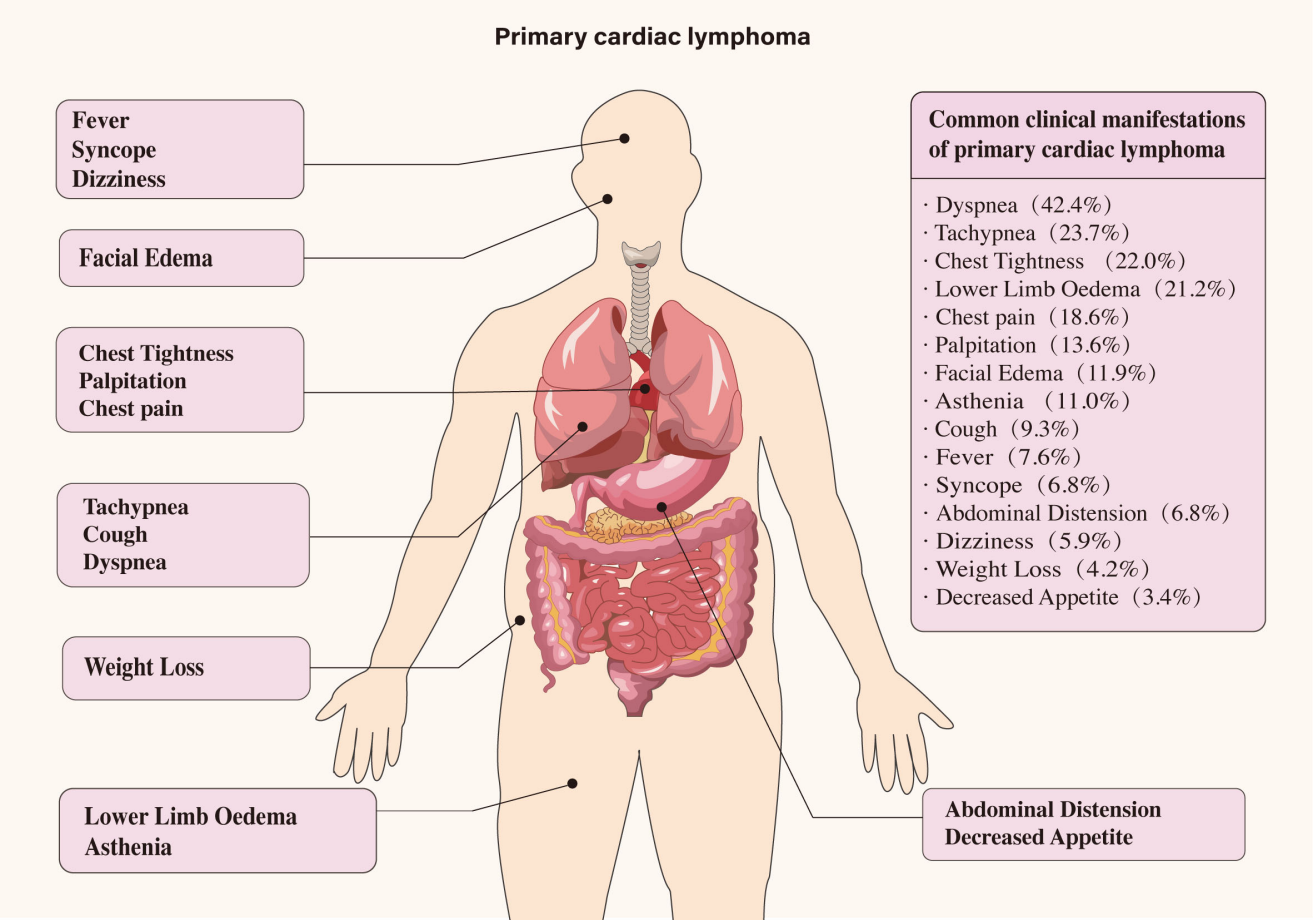
Clinical manifestations of 121 cases of primary cardiac lymphoma.

The primary sites of mass involvement were predominantly the right atrium and ventricle. Other affected areas included the left ventricle, left atrium, and pericardium (**Figure 2**). Clinical symptoms were non-specific and primarily circulatory and respiratory in nature, including dyspnea, chest tightness, chest pain, and tachypnea. Neurological and gastrointestinal symptoms were also observed (details provided in **Figure 3**). Pericardial effusion was noted in 52 patients (43.0%), while pleural effusion was observed in 10 patients (8.2%). Mass sizes varied, with the largest measuring 9.0 × 16.0 × 6.0 cm.

### 3.3 Ancillary tests and type of pathology

Imaging examinations, particularly cardiac color ultrasound, are important in the diagnosis and monitoring of disease progression. Cardiac ultrasound was conducted in 112 out of 121 cases (92.6%), supplemented by computed tomography (CT), magnetic resonance imaging (MRI), and PET-CT, among other modalities. Electrocardiograms and cytological analyses of pericardial and pleural effusions are also instrumental in the diagnostic process; however, pathological confirmation remains essential for the definitive diagnosis of cardiac lymphoma. Of the 112 ultrasound cases, 100 (89.3%) successfully identified the mass, with 37 (33.3%) reporting its size. Others can also clarify the size of the mass through chest CT scans and cardiac MRI. Pericardial effusion was detected in 17 cases (48.6%). Additional ultrasound findings included delineation of mass boundaries, mobility, extent of encroachment, and potential obstruction of valve and vena cava orifices.

Electrocardiographic abnormalities were observed in 44 patients, including atrioventricular block in 19 patients (50%), atrial flutter or atrial fibrillation in 13 patients (29.5%), and complete right bundle branch block in 3 patients (6.8%). Other notable findings included atrial tachycardia, Partial ST-T changes, and escape rhythm.

Pathology confirmed the diagnosis in all 121 patients. 54 cases (44.6%) underwent open chest tumor resection biopsy; 42 cases (34.7%) underwent percutaneous biopsy, including punctures of the mass, lymph node, endocardium, and myocardium. Notably, 6 cases (5.0%) were definitively diagnosed through autopsy; 4 cases (3.4%) were diagnosed via biopsy of metastatic lesions; and 9 cases (7.4%) were assessed diagnostically through pericardial effusion. Pathologically, all 117 cases were classified as non-Hodgkin’s lymphoma; of these, 108 cases (92.3%) were identified as B-cell lymphoma. This category included 83 cases of diffuse large B-cell lymphoma (DLBCL), 2 cases of Burkitt’s lymphoma, a case of plasmablastic lymphoma and 22 cases of unspecified B-cell lymphoma type (**Table 1**).

**Table 1:** Pathologic staging of 121 primary cardiac lymphoma.

Pathologic staging	Number of cases	Percentage (%)
Non-Hodgkin’s lymphoma	117	96.7
B-cell lymphoma	108	89.3
Diffuse large B-cell lymphoma	83	68.6
Plasmablastic lymphoma	1	0.8
Burkitt lymphoma	2	1.7
B-cell lymphoma of unknown type	22	18.2
T-cell lymphoma	7	5.8
Unspecified type of non-Hodgkin’s lymphoma	2	1.7
Hodgkin’s lymphoma	1	0.8
unknown type of lymphoma	3	2.5

### 3.4 Treatment and prognosis

In terms of treatment and prognosis, 54 patients (44.6%) underwent cardiotomy under general anesthesia, while 91 patients (75.2%) received chemotherapy. Among those who received chemotherapy, 78 patients (85.7%) were treated with R-CHOP, E-CHOP, CHOP, R-COP, COP or HOP regimens (R: Rituximab, C: Cyclophosphamide, H: Doxorubicin, O: Vincristine P: Prednisone, E: Etoposide); the specific regimen for 12 patients was not reported. Of the patients who underwent chemotherapy, 74 (80.4%) exhibited effective responses, while 6 (6.5%) had poor outcomes, and 11 (12.1%) outcome was not specified. Additional treatments included heart transplantation *in situ*, radiotherapy, pleural adhesion release, pacemaker implantation, superior vena cava evacuation, pericardial drainage, blood transfusion, anti-infection measures, and other symptomatic interventions (**Table 2**).

**Table 2:** Chart of 121 patients with primary cardiac lymphoma.

Case	Year	Gender/Age	Lump size	Types of lymphoma	ECG	First line treatment		Response	Final outcome	Follow-up (months)
						Surgery	Chemotherapy			
1 (5)	2024	F 66	RA: 70×50mm	DLBCL-nonGCB	N/A	Tumor resection	R-CHOP×5	E	N/A	N/A
2 (6)	2024	F 59	RA: 72×44mm	DLBCL-nonGCB	N/A	Tumor resection	R-CHOP×4	E	Brain metastases after chemotherapy	N/A
3 (7)	2023	F 56	RA: 72 mm × 44 mm	DLBCL-nonGCB	N/A	Tumor resection	N/A	N/A	N/A	N/A
4 (8)	2023	M 81	AV groove: 81 mm × 48 mm	DLBCL-nonGCB	N/A	N/A	R-COP×3	E	N/A	N/A
5 (9)	2023	F 54	N/A	DLBCL-nonGCB	ST, I°AVB	Biopsy only	R-CHOP×5	E	N/A	N/A
6 (10)	2022	F 55	N/A	DLBCL-nonGCB	N/A	N/A	R-CHOP×6	CR	Follow-up 2 years, no recurrence	24
7 (11)	2022	M 56	RA: 88 mm × 63 mm	DLBCL-nonGCB	N/A	Tumor resection	COP×1+R-COP×2+R-GemOX×1+RMA×2+RCHOPE×2+(CHOP+TMZ)×2	NR	Died 1 month after chemotherapy	1
8 (12)	2021	M 67	RA: 52 mm × 50 mm × 44 mm	NHL	N/A	Tumor resection	N/A	N/A	Follow-up 4 months, no recurrence	4
9 (13)	2021	F 45	RA: 55 mm × 44 mm	BCL	N/A	Tumor resection	N/A	N/A	N/A	N/A
10 (14)	2021	M 37	N/A	BCL	N/A	Biopsy only	CDOP×1+(R+CODOXM-A, CODOXM-B, CODOXM-A)×2	CR	N/A	N/A
11 (15)	2021	F 65	ATVL: 30 mm × 16 mm; SL: 44 mm × 24 mm; RA: 25 mm × 9 mm; LV: 35 mm × 39 mm; RV: 25 mm × 35 mm	DLBCL-nonGCB	AT, III°AVB	Tumor resection	CHOP×9	E	Tumour disappears after chemotherapy	N/A
12 (16)	2020	M 64	RA: 5 cm × 3 cm × 3 cm	DLBCL-nonGCB	N/A	Tumor resection	N/A	N/A	Death, 8 months	8
13 (17)	2019	M 47	RA: 59 mm × 35 mm	BCL	AT, III°AVB, JER, Partial ST-T changes	Biopsy only	CHOP×1+R-CHOP×6	E	Follow-up 5 months, no recurrence	5
14 (18)	2019	M 68	N/A	DLBCL	ST,AFL	Palliative resection+ASCT	N/A	E	N/A	N/A
15 (19)	2018	M 48	RA: 7 cm × 8 cm	DLBCL-nonGCB	N/A	Tumor resection	N/A	N/A	N/A	N/A
16 (20)	2017	M 57	N/A	DLBCL	N/A	Biopsy only	CHP×3	E	Death, post-chemotherapy	N/A
17 (21)	2017	M 72	RA: 64×50mm	DLBCL	N/A	Tumor resection	CHOP×8	E	Follow-up 8months, no recurrence	8
18 (22)	2017	F 42	RA: 46 mm × 59 mm	DLBCL-nonGCB	ST, I°AVB, LV, CWR, RAD	Palliative resection	N/A	N/A	N/A	N/A
19 (23)	2016	M 66	RA: 46 mm × 50 mm	DLBCL-GCB	N/A	Biopsy only	CHOP×1+R-CHOP×5+R-GDP×1	NR	Tumour metastasis after chemotherapy	N/A
20 (24)	2016	F 46	RA: 63 mm × 37 mm	DLBCL	N/A	Tumor resection	Unknown	E	Follow-up 5 months, no recurrence	5
21 (25)	2016	F 55	RA: 38 mm × 40 mm	DLBCL	N/A	Tumor resection	N/A	N/A	N/A	N/A
22 (26)	2016	M 71	N/A	DLBCL-nonGCB	N/A	Tumor resection	CHOP×3	E	N/A	N/A
23 (27)	2016	F 70	RA: 60 mm × 47 mm	NHL	N/A	Tumor resection	N/A	N/A	N/A	N/A
24 (28)	2015	M 67	RA: 77 mm × 50 mm	DLBCL	N/A	Tumor resection	N/A	N/A	N/A	N/A
25 (29)	2015	M 33	RA: 34 mm × 27 mm × 30 mm	DLBCL	N/A	N/A	CHOP×1	N/A	N/A	N/A
26 (30)	2014	F 63	RA: 19 mm × 18 mm RV: 27 mm × 19 mm	DLBCL	N/A	Biopsy only	N/A	N/A	Died of multi-organ failure 9 months after surgery	9
27 (31)	2014	F 68	Right atrium and lateral right ventricle: 67×55mm	BCL	N/A	Biopsy only	N/A	N/A	N/A	N/A
28 (32)	2024	M 75	RA: 34×26mm、44×83mm	DLBCL	N/A	Biopsy only	CHOP×1+R-CHOP×3	CR	Follow-up 6months, no recurrence	6
29 (33)	2024	F 72	N/A	DLBCL-nonGCB	III°AVB	Biopsy only	R-CHOP	NR	Died 6 weeks after admission	1.5
30 (34)	2024	F 65	N/A	DLBCL	AFL	N/A	N/A	N/A	Died soon after admission	0
31 (35)	2024	M 60	N/A	DLBCL-GCB	N/A	Biopsy only	pola-R-CHP	E	N/A	N/A
32 (36)	2023	F 46	RA: 65×53mm	DLBCL	N/A	Biopsy only	DA-R-EPOCH	E	N/A	N/A
33 (37)	2023	F >60	N/A	DLBCL	Biopsy only	N/A	DA-EPOCH-R×1+DA-EPOCH-R×5	CR	Still CR at 6 months follow-up after chemotherapy	6
34 (38)	2023	M 75	RV: 92×33mm	DLBCL	N/A	Biopsy only	Unknown	E	N/A	N/A
35 (39)	2022	F 67	RA: 48×32mm	DLBCL	N/A	Tumor resection	N/A	N/A	Died 5 months after surgery	5
36 (40)	2022	M 65	RA: 35×37mm	DLBCL	N/A	N/A	N/A	N/A	Died 34 days after admission	1
37 (41)	2022	F 47	N/A	TCL	III°AVB	Biopsy only	MTX+CHOP	CR	N/A	N/A
38 (42)	2022	M 65	N/A	DLBCL	N/A	Palliative resection	Unknown	E	No recurrence 3 months after surgery	3
39 (43)	2022	M 54	N/A	DLBCL-GCB	III°AVB	Biopsy only	CHOP	E	Follow-up 6 months, no recurrence	6
40 (44)	2022	F 77	N/A	DLBCL-nonGCB	ST	Biopsy only	R×2+R_2_×2+R-miniCHOP×1+R^2^-miniCHOP×5	E	Follow-up 5 months, no recurrence	5
41 (45)	2022	M 70	RV: 25×18mm	BL	N/A	Biopsy only	R-HOP+IT MTX×1+R-EPOCH×1+R-EPOCH×2+ IT MTX×1	E	Died 172 days after admission	6
42 (46)	2022	M 70	N/A	DLBCL-nonGCB	III°AVB	Biopsy only	DA-EPOCH-R×5	CR	Follow-up 6 months, no recurrence	6
43 (47)	2021	F 92	N/A	DLBCL	AF	N/A	N/A	N/A	Died 1 month after diagnosis	1
44 (48)	2021	M 70	RA、RV: 44×56mm	DLBCL	N/A	Tumor resection	Unknown	E	Follow-up 6 months, no recurrence	6
45 (49)	2021	M 65	N/A	DLBCL	N/A	Biopsy only	R-COP×3	E	Progress towards the end of chemotherapy	N/A
46 (50)	2021	M 55	N/A	BCL	N/A	N/A	R-CHOP+R-CHASE	CR	Still CR at 1 year follow-up	12
47 (51)	2021	F 55	CS: 13.5cm^3^	DLBCL	SVT	Tumor resection	R-CHOP×6	E	Follow-up 5 months, no recurrence	5
48 (52)	2021	M 61	N/A	DLBCL	N/A	Biopsy only	R-CHOP	N/A	N/A	N/A
49 (53)	2020	M 68	RV: 26×29mm	DLBCL	N/A	N/A	R-CHOP×6	CR	CR after chemotherapy	N/A
50 (54)	2020	F 59	RA: 85×50mm	BCL	III°AVB	Tumor resection	R-CHOP	E	Brain metastases after surgery	N/A
51 (54)	2020	M 53	LA: 40mm(W)	BCL	N/A	Tumor resection	R-CHOP	E	Follow-up 7 months, no recurrence	7
52 (55)	2020	F 68	N/A	DLBCL	AF	N/A	R-CHOP×6	E	N/A	N/A
53 (56)	2020	M 67	LV: 35×48mm	DLBCL	CRBBB	Tumor resection	R-CHOP×6	E	Surviving 1 year after chemotherapy	12
54 (57)	2020	F 14	RA: 46mm(W)	HL	N/A	Biopsy only	AVEPC	E	N/A	N/A
55 (58)	2019	M 64	RA、RV: 100×100×50mm	DLBCL-nonGCB	AF	Palliative resection	R-CHOP×5+DHAP×2	NR	Still CR at 4 years follow up	48
56 (59)	2019	M 46	RA: 50×35mm	DLBCL	III°AVB,AFL	Palliative resection+ASCT	R-CHOP×6+R-EPOCH×1	CR	Still CR at 1 years follow up	12
57 (60)	2019	M 69	N/A	BCL	N/A	N/A	R-CHOP	N/A	N/A	N/A
58 (61)	2019	M 38	N/A	TCL	III°AVB	Biopsy only	N/A	N/A	N/A	N/A
59 (62)	2019	M 48	RA: 70×80mm	BCL	N/A	Tumor resection	Unknown	N/A	Died 4 months after surgery	4
60 (63)	2019	F 63	RA: 83×64×53mm	DLBCL-nonGCB	ST	Biopsy only	R-CHOP×7	E	Surviving 20 months after diagnosis	20
61 (64)	2019	M 82	RA: 86×47mm	DLBCL	III°AVB	Biopsy only	R-CHOP×6	E	N/A	N/A
62 (65)	2019	M 28	N/A	DLBCL	N/A	Biopsy only	R-CHOP×8	CR	Follow-up 15 months, no recurrence	15
63 (66)	2019	M 55	N/A	DLBCL	N/A	Tumor resection	N/A	N/A	Died 1 month after diagnosis	1
64 (66)	2019	F 61	N/A	DLBCL	N/A	Tumor resection	R-CHOP×4	E	Follow-up 18 months, no recurrence	18
65 (67)	2019	M 51	RA: 34×23mm	Unknown	N/A	Tumor resection	Unknown	NR	Died 2 days after surgery	0
66 (68)	2019	F 64	N/A	BL	III°AVB	Tumor resection	CHOP	PR	PR 7 weeks after surgery	1.75
67 (69)	2018	M 79	RA: 25×40mm	DLBCL	N/A	Palliative resection	R-CHOP×8	CR	Still CR 456 days after surgery	15
68 (70)	2018	M 57	RA: 60×45mm	BCL	N/A	Tumor resection	R-CHOP	N/A	N/A	N/A
69 (71)	2018	M >40	RA、RV: 100×60mm	DLBCL	N/A	N/A	N/A	N/A	sudden death	0
70 (72)	2018	M 64	N/A	DLBCL	N/A	Biopsy only	Unknown	N/A	N/A	N/A
71 (73)	2018	M 79	N/A	DLBCL	N/A	Palliative resection	R-CHOP	E	Died 14 days after chemotherapy	0.5
72 (74)	2018	M 87	N/A	DLBCL	N/A	N/A	N/A	N/A	Died 9 days after admission	0.25
73 (75)	2018	M 49	RA: 135×103mm	Unknown	N/A	Biopsy only	Unknown	E	N/A	N/A
74 (76)	2018	F 78	N/A	DLBCL	N/A	Biopsy only	R-CHOP	N/A	N/A	N/A
75 (77)	2018	M 37	RA: 84×74×76mm	DLBCL	N/A	Tumor resection+ASCT	R-CHOP×8+R-ESHAP×2	E	Follow-up 4 years, no recurrence	48
76 (78)	2018	M 58	N/A	DLBCL-nonGCB	AF	Tumor resection	R-CHOP	N/A	Died 26 days after surgery	1
77 (79)	2017	F 83	N/A	DLBCL	AF	Palliative resection	Unknown	E	Follow-up 1 year, no recurrence	12
78 (80)	2017	F 52	N/A	BCL	N/A	Tumor resection	N/A	N/A	N/A	N/A
79 (81)	2017	F 62	Reri: 90×160×60mm	TCL	N/A	Biopsy only	CHOP	N/A	N/A	N/A
80 (82)	2017	M 35	LV: 10×20mm	BCL	N/A	Tumor resection	R-CHOP×6	CR	N/A	N/A
81 (83)	2017	M 59	RA: 95mm(max)	BCL	N/A	Biopsy only	R-COMP	E	N/A	N/A
82 (84)	2017	F 65	RA: 66×57mm	DLBCL	SSS	Tumor resection	R-CHOP×8	E	Follow-up 11 months, no recurrence	11
83 (85)	2017	F 32	N/A	TCL	AF	N/A	N/A	N/A	N/A	N/A
84 (86)	2017	M 73	RA: 83×52mm	DLBCL	N/A	Tumor resection	CHOP	N/A	Died 6 months after surgery	6
85 (86)	2017	F 27	N/A	DLBCL	AF	Tumor resection	R-CHOP×5	CR	Follow-up 20 months, no recurrence	20
86 (87)	2017	F 11	N/A	TCL	V	Tumor resection	MTX-CHOP	E	Died 2 months after surgery	2
87 (88)	2017	F 64	RA: 66×57mm	DLBCL	N/A	Tumor resection	N/A	N/A	N/A	N/A
88 (89)	2016	M 67	N/A	DLBCL	N/A	Tumor resection	R-CHOP×6	E	Follow-up 5 months, no recurrence	5
89 (90)	2016	F 53	N/A	BCL	AF	Biopsy only	CP×1+HO×1+R-HO×3	E	No relapse 2 years after the start of treatment	24
90 (91)	2016	F 61	RA: 40×50×60mm	DLBCL	N/A	Tumor resection	N/A	N/A	Died 2 weeks after surgery	0.5
91 (91)	2016	M 74	N/A	DLBCL	AF	Biopsy only	N/A	N/A	Died 1 week after biopsy	0.25
92 (92)	2016	M 54	N/A	BCL	N/A	Biopsy only	R-CHOP	E	N/A	N/A
93 (93)	2016	M 17	RAA: 45×61mm	TCL	N/A	Tumor resection	MTX-P	E	N/A	N/A
94 (94)	2016	F 67	N/A	BCL	N/A	Biopsy only	N/A	N/A	N/A	N/A
95 (95)	2016	M 65	Reri: 100×70mm	DLBCL	AF	Biopsy only	N/A	N/A	Died soon after diagnosis	0
96 (96)	2016	M 26	N/A	TCL	N/A	Biopsy only	CVP	E	Died 6 months after diagnosis	6
97 (97)	2015	M 58	RA: 67×55mm	DLBCL	N/A	Palliative resection	R-CHOP×6	E	N/A	N/A
98 (98)	2015	M 62	N/A	DLBCL	N/A	Biopsy only	R-CHOP	E	N/A	N/A
99 (99)	2015	M 57	RA: 56×35mm	PBL	CRBBB	Tumor resection	DA-EPOCH×6	CR	Still CR 2.5 years after starting treatment	15
100 (100)	2015	F 73	RA: 60×75mm	DLBCL	N/A	Palliative resection	CHOP×6	E	No recurrence 13 years after surgery	156
101 (101)	2015	F 48	N/A	DLBCL	N/A	Fontan	Unknown	E	N/A	N/A
102 (101)	2015	F 64	RA: 100×72×80mm	DLBCL-nonGCB	N/A	Fontan	N/A	E	Died soon after surgery	0
103 (102)	2015	M 71	RV: 30×60mm	DLBCL	III°AVB	Biopsy only	R-CHOP×3+R-CEOP×3	CR	Follow-up 2 years, no recurrence	24
104 (103)	2015	M 65	N/A	BCL	AF	Biopsy only	COP	CR	CR after 6 months of chemotherapy	6
105 (104)	2015	M 52	N/A	BCL	N/A	Biopsy only+ASCT	R-Hyper-CVAD×3+R-ICE×2	NR	Recurrence of death at 74 days follow-up	2.5
106 (105)	2015	M 60	N/A	DLBCL	N/A	Palliative resection	EPOCH-R_2_	E	N/A	N/A
107 (106)	2015	M 62	N/A	BCL	N/A	Biopsy only	R-CHOP×6	CR	Follow-up 8 months, no recurrence	8
108 (107)	2015	M 79	N/A	DLBCL	CRBBB	Biopsy only	CHOP×1+R-CHOP×7	E	N/A	N/A
109 (108)	2015	F 35	N/A	DLBCL-nonGCB	N/A	Biopsy only+ASCT	R-CHOP×3	CR	N/A	N/A
110 (109)	2015	M 65	N/A	Unknown	N/A	Biopsy only	N/A	N/A	N/A	N/A
111 (110)	2015	M 71	RA: 83mm(W)	BCL	N/A	N/A	R-CHOP×8	E	N/A	N/A
112 (111)	2014	F 76	RA: 60×45mm	DLBCL	III°AVB	Biopsy only	R-THP-COP	E	Stable 15 months after admission	15
113 (112)	2014	M 43	PV: 21mm(W)	DLBCL	N/A	Biopsy only	Unknown	E	Follow-up 6 months, no recurrence	6
114 (113)	2014	M 58	RA: 60×90×70mm	DLBCL	N/A	Biopsy only	R-CHOP	E	Follow-up 12 months, no recurrence	12
115 (114)	2014	F 55	N/A	DLBCL	N/A	Biopsy only	R-CHOP	E	Follow-up 4 months, alive	4
116 (115)	2014	M 70	N/A	BCL	III°AVB	Tumor resection	N/A	N/A	N/A	N/A
117 (116)	2014	F 55	N/A	DLBCL	N/A	Biopsy only	R-CHOP	E	N/A	N/A
118 (117)	2014	F 60	N/A	DLBCL	N/A	Biopsy only	R-COP	E	N/A	N/A
119 (118)	2014	M 44	N/A	BCL	N/A	Biopsy only	R-CHOP	N/A	Died 4 months after diagnosis	4
120 (119)	2014	F 70	RA: 80mm(W)	DLBCL	N/A	Tumor resection	R-CHOP×6	N/A	N/A	N/A
121 (120)	2014	M 62	RA: 30×45mm	DLBCL	N/A	Tumor resection	R-CHOP×6	E	N/A	N/A

F, female; M, male; yrs, years; RA, Right Atrium; RV, Right Ventricle; LA, Left Atrium; LV, Left Ventricle; W, width; SL, septal leaflet; ATVL, Anterior tricuspid valve leaflet; LV, Low Voltage; CS, Coronary Sinus; Peri, Pericardium; DLBCL, diffuse large B-cell lymphoma; BL, Burkitt’s lymphoma; nGCB, non-Germinal Center B-cell-like; GCB, Germinal Center B-cell-like; BCL, B-cell lymphoma; NHL, Non-Hodgkin Lymphoma; HL, Hodgkin Lymphoma; TCL, T-cell lymphoma; PBL, Plasmablast Lymphoma; ST, Sinus tachycardia; AVB, AV block; AT, Atrial tachycardia; JER, junctional escape rhythm; CWR, Clockwise Rotation of the Electrocardiogram; RAD, right axis deviation; AFL, Atrial Flutter; AF, Atrial Fibrillation; CRBBB, Complete Right Bundle Branch Block; SVT, Supraventricular Tachycardia; SSS, sick sinus syndrome; RAA, Right Atrial Appendage; PV, Pulmonary Valve; ASCT, Autologous Stem Cell Transplantation; R, Rituximab; C, Cyclophosphamide; H, Doxorubicin; O, Vincristine; P, Prednisone; GEMOX, Gemcitabine + Epirubicin + Methotrexate + Oxaliplatin; TMA, Thiotepa + Methotrexate + Actinomycin D; CODOXM-A, cyclophosphamide + vincristine + doxorubicin + hydrochloride + liposome + methotrexate (with temozolomide replacing methotrexate in regimen A); CODOXM⁃B, isocyclophosphamide + etoposide + cytarabine; R-GDP, rituximab + gemcitabine + cisplatin + dexamethasone; pola, Polarubicinide; DA-R-EROCH, Rituximab + Etoposide + Vincristine + Doxorubicin + Cyclophosphamide + Prednisone; MTX, Methotrexate; R2, Rituximab + Lenalidomide; ITMTX, Ifosfamide + Thiotepa + Methotrexate + Etoposide; R-CHASE, Rituximab + Cyclophosphamide + Hydroxydaunorubicin + Oncovin + Etoposide + Dexamethasone; AVEPC, Doxorubicin + Vincristine + Etoposide + Prednisone + Cyclophosphamide; DHAP, Dexamethasone + High-dose Cytarabine + Cisplatin; R-ESHAP, Rituximab + Etoposide + Solu-Medrone + High-dose Cytarabine + Cisplatin; R-COMP, Rituximab + Cyclophosphamide + Liposomal Doxorubicin + Vincristine + Prednisone; CVP, Cyclophosphamide + Vincristine + Prednisolone; MTX-P, Methotrexate + Procarbazine; R-Hyper-CVAD, Rituximab + Hyperfractionated Cyclophosphamide + Vincristine + Doxorubicin + Dexamethasone; R-ICE, Rituximab + Ifosfamide + Carboplatin + Etoposide; R-THP-COP, Rituximab + Tetrahydropyranyl Adriamycin + Cyclophosphamide + Vincristine + Prednisolone; E, Effective; CR, Complete Response; PR, Partial Response; NR, Non-Response;

“N/A” means not mentioned in the text.

Regarding patient prognosis, only patients with available prognostic information (excluding those who received ASCT) were included in this analysis, totaling 59 cases. A total of 8 patients (13.3%) did not undergo any treatment, with a median survival of only 1 week; 5 patients (8.3%) received tumor resection only, of whom 2 died within 30 days, with a 30-day mortality rate of 40%; 22 patients (36.7%) received chemotherapy only, of whom 4 (18.2%) died, with a median survival time of 6 months; 18 patients survived, with a median follow-up time of 7 months by the date of follow-up; 24 patients (40.0%) received both tumor resection and chemotherapy, of whom 7 (29.2%) died, with a median survival time of 1 month; 17 patients survived, with a median follow-up time of 11 months by the date of follow-up.

## 4 Case report

A 73-year-old female patient was admitted to the hospital on November 6, 2020, reporting chest tightness after physical activity, which had persisted for over a month. The patient had a four-year history of thrombocytosis, which was managed with oral hydroxyurea. Upon examination, her vital signs were as follows: temperature 36.1°C, heart rate 110 beats/min, respiratory rate 12 breaths/min, blood pressure 94/60 mmHg, peripheral capillary oxygen saturation (SpO_2_) 98%. The patient was conscious and exhibited steady breathing and responsiveness, and had an Eastern Collaborative Oncology Group performance status of 1. The physical examination showed no significant abnormalities, except for coarse breath sounds detected in both lungs.

**Figure 4:**
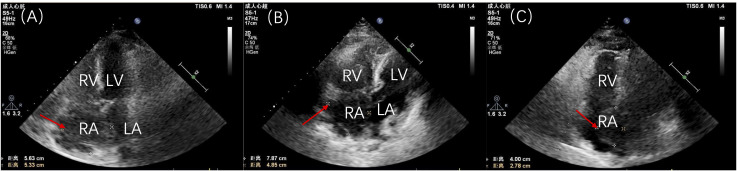
Patient’s cardiac ultrasound findings. **(A)** Initial cardiac ultrasound demonstrating a large tumor occupying nearly the entire right atrium. **(B)** Preoperative cardiac ultrasound review indicating an increase in size of the hypoechoic mass in the right atrium, now measuring approximately 75 mm × 50 mm and attached to the atrial septum. **(C)** Postoperative cardiac ultrasound revealing a hypoechoic mass in the right atrium, measuring approximately 40 mm × 28 mm. RV, right ventricle; RA, right atrium; LV, left ventricle; LA, left atrium.

Initial laboratory tests indicated leukocyte levels at 11.90×10^9^/L, hemoglobin at 135.0 g/L, neutrophils at 7.78×10^9^/L, platelets at 265×10^9^/L, and a monocyte percentage of 18.4%. C-reactive protein was measured at 18.3 mg/L, lactate dehydrogenase at 360.0 U/L, while liver and kidney function tests, coagulation parameters, and electrolyte levels remained within normal ranges. Cardiac color ultrasound revealed enlargement of both the right atrium and ventricle, with the right ventricular end-diastolic transverse diameter measuring 42 mm. A hyperechoic mass, approximately 56 mm × 53 mm, was identified in the right atrium, indicating a possible mucinous tumor. This mass partially shifted toward the right ventricular side through the tricuspid valve during diastole. This movement resulted in accelerated blood flow at the tricuspid opening. Additionally, a small amount of pericardial effusion was observed (**Figure 4A**). A follow-up ultrasound on November 11, 2020, demonstrated an increase in the size of the hypoechoic mass in the right atrium, which now measured approximately 75 mm × 50 mm (**Figure 4B**). This mass, attached to the atrial septum, exhibited increased mobility and intermittently obstructed the tricuspid valve orifice during cardiac cycles. The patient’s family, considering their financial situation, did not proceed with PET-CT for tumor staging assessment.

**Figure 5:**
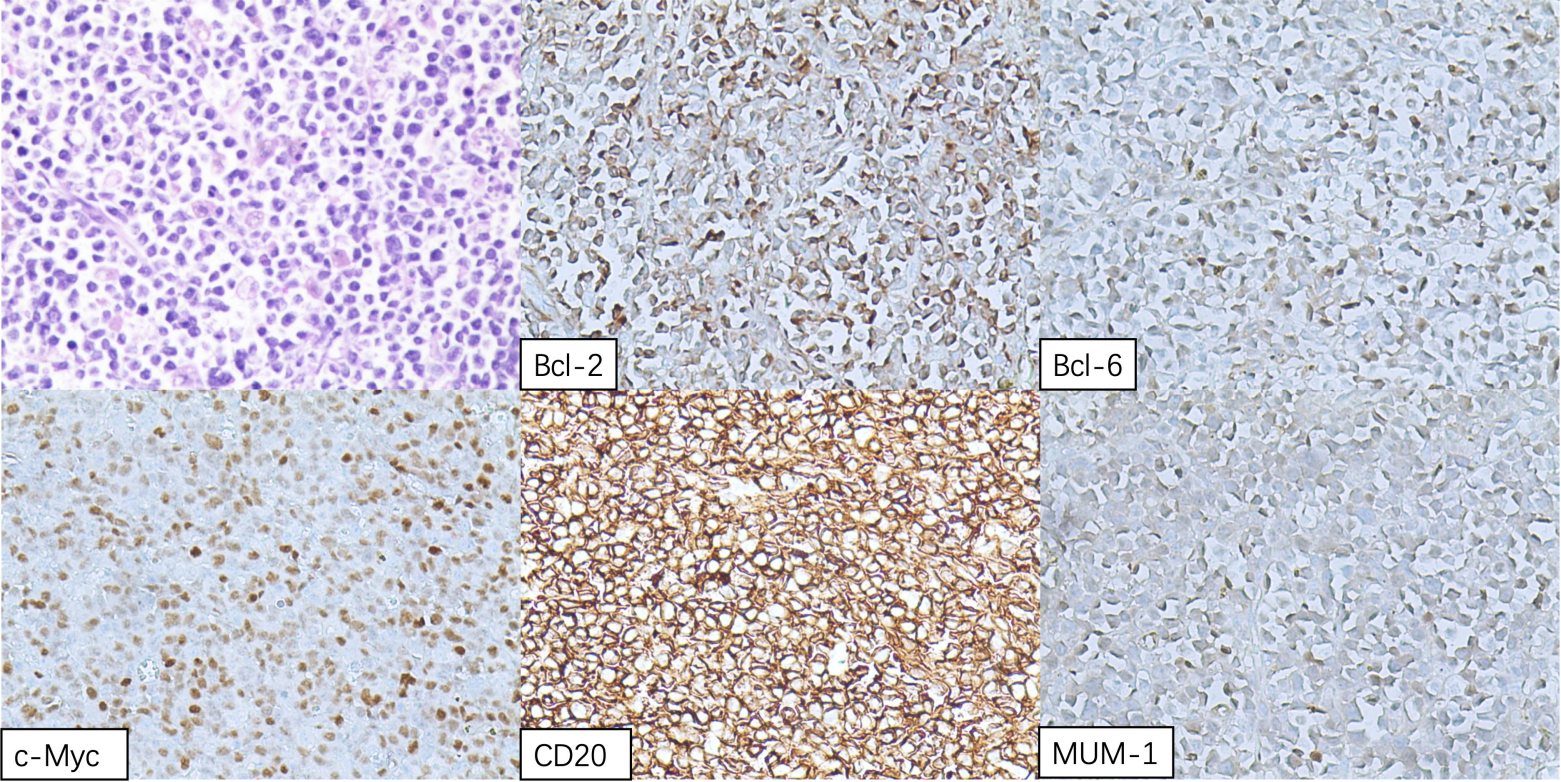
Hematoxylin and eosin (H&E) staining of the right atrial mass displaying sheets of lymphomatous large cells.

The patient underwent resection of a cardiac tumor under general anesthesia on November 16. Intraoperative findings revealed multiple cauliflower-shaped masses in the right atrium, varying in size, with the largest measuring approximately 8 cm × 4.5 cm, extending to the tricuspid valve and interatrial septum. A palliative resection of the tumor was performed. Postoperative pathology identified the masses as diffuse large B-cell lymphoma (non-germinal center origin), confirmed through immunohistochemical profiling: CD3 (-), CD20 (+), CD21 (-), Ki67-MIB1 (70%), CD30 (few +), Bcl-2 (+), CK (-), CD10 (-), Bcl-6 (+), MUM-1 (+), c-myc (50%), P53 (few +), Pax-5 (+), Cyclin D1 (-), CD5 (-), and negative EBER *in situ* hybridization (**Figure 5**).

The final diagnosis was cardiac diffuse large B-cell lymphoma (non-germinal center origin), according to the 2015 World Health Organization (WHO) Classification of Cardiac and Pericardial Tumors (1). Considering the patient’s age, the hematology department recommended low-dose chemotherapy. However, her family, taking into account the patient’s age and their own financial situation, decided to refuse chemotherapy. The postoperative cardiac ultrasound revealed findings consistent with changes typically observed in right and left atrial occupying lesions. A hypoechoic mass, measuring approximately 40 mm × 28 mm, was present in the right atrium, extending from the base to the upper part of the interatrial septum, with a protrusion into the left atrium measuring approximately 22 mm × 16 mm (**Figure 4C**). After recovery, the patient was discharged on November 20, 2020. Unfortunately, she passed away on April 20, 2021, without seeking further medical consultations during this period.

This case aligns with several characteristics of PCL described in the existing literature, including its demographic predilections, tumor size, common sites of involvement, and histological subtypes. The patient’s presenting symptoms of chest discomfort and exertional dyspnea are consistent with the commonly reported symptoms of PCL, such as chest pain and dyspnea. Moreover, the brief interval between surgical intervention and the patient’s demise highlights the highly aggressive nature, rapid progression, and high mortality rate associated with PCL. This also underscores the limitations of palliative surgical resection as a standalone treatment strategy. However, this case exhibits unique clinical features that may be influenced by individual factors and disparities in access to medical resources. Notably, the patient had a four-year history of primary thrombocythemia and was undergoing treatment with hydroxyurea, an unusual finding among PCL patients. Additionally, the patient demonstrated an exceptionally rapid tumor growth rate, as evidenced by a marked increase in tumor volume between the initial echocardiogram and subsequent follow-up examinations. This suggests a more aggressive disease course than what is typically observed in PCL cases. These distinct features warrant further investigation to better understand their implications for disease progression and management.

## 5 Discussion

PCL is an exceedingly rare malignancy, predominantly presenting as cardiac and/or pericardial tumor tissue or as myocardial infiltration of lymphomas, which results in cardiac symptoms. Representing only 1% of primary cardiac malignancies and 0.5% of all extranodal lymphomas (121). This article summarizes the clinical presentation, ancillary investigations, diagnosis, treatment and prognosis from a decade of case reports and describes a particular PCL patient.

As the second most prevalent primary cardiac malignancy, PCL has a dire prognosis without treatment, often limited to just a few months. Therefore, prompt and accurate diagnosis and treatment are crucial. However, the non-specific clinical manifestations and auxiliary examinations present challenges for early diagnosis. Clinically, the approach to pathological biopsy should evolve from non-invasive to invasive methods to achieve efficient and cost-effective diagnosis.

The incidence of B symptoms in patients with PCL is notably low. Instead, the condition commonly presents with symptoms such as shortness of breath, chest tightness, and lower limb edema. These may occasionally be accompanied by additional symptoms, including loss of appetite, anxiety, and chest pain, further contributing to the clinical complexity of PCL. Besides, it can cause a variety of arrhythmias, with atrial fibrillation, atrial flutter, and third-degree atrioventricular block being the most common. Lesions are predominantly located in the cardiac and pericardial regions, with the right atrium being the most commonly involved site. This finding aligns with our study, where 86.1% of cases involved the right atrium. Notably, 8 patients (6.6%) presented to the hospital with syncope, which was associated with a poor prognosis. Among these, 2 patients experienced disease progression, and 3 succumbed to the condition within 4 months. These findings underscore the importance of considering this disease in the differential diagnosis when evaluating comatose patients. Clinicians are advised to maintain a high index of suspicion to ensure timely identification and management. Overall, the symptoms of PCL are subtle at first, becoming more pronounced as the lymphoma progresses, often leading to late-stage diagnoses.

The need for new diagnostic methods is paramount, even as the accuracy of existing imaging examinations improves. Commonly utilized clinical auxiliary examinations include electrocardiography (ECG), transthoracic echocardiography (TTE), transesophageal echocardiography (TEE), cardiac computed tomography (CT), cardiac magnetic resonance (CMR) and FDG-PET. Echocardiography, as an adjunctive test for the initial diagnosis of cardiac tumors, is able to dynamically observe the location, size, morphology, mobility of cardiac tumors and their relationship with the surrounding tissues, to find the presence of hypoechoic masses and to detect associated pericardial effusions. TTE is not as sensitive as TEE in identifying cardiac tumors, and a retrospective study by Ceresoli (2) et al. demonstrated that TTE detected a cardiac tumors, whereas TEE provides better visualization of cardiac structures, especially those away from the chest wall (63). Therefore, we prefer TEE for the initial diagnosis of cardiac tumors. The use of CT and CMR imaging in the diagnosis of PCL has been increasing steadily in recent years. Asadian (122) et al. have highlighted that these imaging modalities, with their ability to employ various parameter settings and provide excellent soft tissue contrast, are valuable tools for characterizing PCL. They allow for detailed assessment of tumor characteristics, differentiation between benign and malignant lesions, and evaluation with or without contrast enhancement. This capability is particularly beneficial in distinguishing PCL from cardiac thrombus, thereby aiding in the differential diagnosis. Furthermore, PET-CT offers a non-invasive approach to assess the metabolic activity of tumors using fluorodeoxyglucose (FDG). Studies have demonstrated that PCL exhibits higher standardized uptake values (SUVs) and larger metabolic tumor volumes on PET imaging compared to primary cardiac sarcoma (PCS) (123). Additionally, PET-CT provides a clearer delineation of tumor invasion, facilitating safer biopsy procedures and guiding subsequent surgical interventions.

The treatment options for PCL include surgical resection, chemotherapy, radiotherapy, and hematopoietic stem cell transplantation, etc. Chemotherapy plays a central role in the treatment of PCL, especially the R-CHOP regimen, which has been widely utilized since 2010. This regimen has notably enhanced the progression-free survival of patients with non-Hodgkin’s B-cell lymphoma (the most common type of PCL). In our study, 44.6% of patients underwent tumor removal surgery, while 76.0% opted for chemotherapy, and only 32.2% combined these approaches. An analysis conducted by Yin et al. (124) using the SEER database revealed that surgical intervention did not improve survival outcomes in patients with PCL, with chemotherapy identified as the sole effective treatment modality (65, 125, 126). However, our study demonstrated that patients who underwent combined surgical resection and chemotherapy exhibited superior survival outcomes compared to those who received surgery or chemotherapy alone. Moreover, for hemodynamically unstable patients, surgical intervention remains a critical and urgent treatment to stabilize their condition (127). Emerging therapeutic options, such as autologous hematopoietic stem cell transplantation (auto-HSCT), allogeneic hematopoietic stem cell transplantation (allo-HSCT), and molecularly targeted therapeutic agents, are showing promise in improving survival rates and prognosis for PCL patients. In parallel, supportive care plays a vital role in the comprehensive management of PCL. This includes symptomatic treatment, nutritional support, palliative care, and psychological counseling. For instance, while the Fontan procedure does not directly treat PCL, it enhances cardiac function, improves the patient’s quality of life, and increases the likelihood of long-term survival, thereby facilitating opportunities for subsequent follow-up treatments.

The prognosis for PCL is generally unfavorable. According to statistics from the SEER database, which included 184 cases of PCL, the 1-year, 3-year, and 5-year survival rates were 59%, 41%, and 34% (128), respectively. More than half of the patients had an overall survival (OS) of less than 3 years, or even shorter. Although there is no uniform conclusion on the treatment of PCL at home and abroad, the R-CHOP regimen is still the most important treatment for PCL because of its remarkable efficacy in B-cell lymphoma. In the case presented, a large, mobile PCL mass that obstructed the tricuspid valve and extended into the left atrium was surgically removed. However, the absence of postoperative chemotherapy led to a poor prognosis, and the patient unfortunately passed away within two months after being discharged from the hospital. This underscores the significance of chemotherapy in the treatment process.

In conclusion, it is crucial to consider cardiac lymphoma as a differential diagnosis when patients present with unexplained cardiac abnormalities, such as heart failure, atrial fibrillation, pericardial effusion, or superior vena cava syndrome, particularly if cardiac ultrasound identifies an intracardiac mass accompanied by unexplained fever. Advancements in imaging modalities, including cardiac CT, CMR, and PET-CT, play a pivotal role in characterizing the mass, assessing its benign or malignant nature, and determining the disease stage. These tools not only guide subsequent biopsy and potential surgical intervention but also enable dynamic adjustments to diagnostic and therapeutic strategies, thereby minimizing the risk of misdiagnosis or delayed diagnosis. Once a diagnosis of cardiac lymphoma is confirmed, individualized clinical judgment is essential to evaluate the need for surgical tumor resection to alleviate cardiac dysfunction. This approach, combined with chemotherapy, can significantly enhance treatment efficacy and improve patient outcomes.

## Data Availability

The original contributions presented in the study are included in the article/supplementary material. Further inquiries can be directed to the corresponding authors.
